# Bosutinib: A Novel Src/Abl Kinase Inhibitor for Chronic Myelogenous Leukemia

**DOI:** 10.6004/jadpro.2013.4.6.8

**Published:** 2013-11-01

**Authors:** Alison Steinbach, Stephen M. Clark, Amber B. Clemmons

**Affiliations:** From Georgia Regents Medical Center and University of Georgia College of Pharmacy, Augusta, Georgia

Chronic myeloid leukemia (CML) is a myeloproliferative disorder characterized by the disease-defining translocation between chromosomes 9 and 22 resulting in the Bcr-Abl tyrosine kinase fusion protein, termed the Philadelphia (Ph+) chromosome [t(9;22)(q34;q11)] (Boschelli, Arndt, & Gambacorti-Passerini, 2010). The introduction of targeted therapy with imatinib mesylate (Gleevec), a tyrosine kinase inhibitor (TKI) effective against Bcr-Abl, significantly improved outcomes for patients with CML.

In the IRIS (International Randomized Study of Interferon and STI-571) trial, imatinib showed significantly higher rates of hematologic and complete cytogenetic response (CCyR), lower risk of progression to advanced disease states (accelerated phase [AP] and blast phase [BP]), and increased progression-free survival (PFS) when compared with interferon-alfa plus cytarabine, the previous standard of care (see Table 1; O’Brien et al., 2003). An 8-year follow- up of the IRIS trial reported a CCyR rate of 83% and an estimated overall survival (OS) rate of 93%. Despite these positive results, 17% of patients treated with imatinib did not achieve a CCyR, approximately 15% of patients who achieved a CCyR eventually lost their response, and an additional 4% to 8% of patients were intolerant to imatinib (Deininger et al., 2009).

Since its approval in 2001, imatinib has been the standard treatment for patients with chronic-phase (CP-CML) disease; however, clinical trial data have demonstrated both imatinib resistance and intolerance due to toxicities. The incidence of resistance to imatinib in untreated CP-CML is approximately 4% per year, yet it was found to be much higher in patients in AP (40%) and BP (90%; Santos et al., 2011).

To address these issues, second-generation inhibitors of Bcr-Abl—dasatinib (Sprycel) and nilotinib (Tasigna)—have been developed. Both of these second-generation TKIs are more potent than imatinib in vitro and have demonstrated efficacy in patients with resistance or intolerance to imatinib (Kantarjian et al., 2011). Unfortunately, resistance to second- generation TKIs can also arise from Bcr-Abl amplification, low bioavailability of active drug, and point mutations within the protein sequence. Alternative treatment options may offer benefit to those patients with intolerance and/or resistance to previous treatment with imatinib, dasatinib, or nilotinib.

Bosutinib (Bosulif) is a new second-generation TKI targeting the Bcr-Abl protein (Puttini et al., 2006). On September 4, 2012, the US Food and Drug Administration (FDA) approved bosutinib for the treatment of CP, AP, or BP Ph+ CML in adults with resistance or intolerance to prior therapy (FDA, 2012).

## Molecular Target Src/Abl

Bosutinib, previously known as SKI-606, is an orally active, dual inhibitor of the Src and Abl tyrosine kinases, both of which are thought to be involved in the development of malignancies (Puttini et al., 2006). Src is a protein kinase that modulates intracellular signal transduction pathways involved in the control of cell growth, differentiation, and migration. Studies have shown that Bcr-Abl domains interact and activate the Lyn and Hck Src kinases through phosphorylation and direct binding. This dual inhibition may be useful in overcoming the onset of some types of resistance that frequently appear in the advanced phases of CML. Due to sequence homologies and structural similarities among the Src and Abl binding domains, ATP inhibitors targeting the inactive conformation of Src are also potent Abl inhibitors (Hu et al., 2004).

Unlike imatinib and dasatinib, bosutinib does not significantly inhibit KIT or PDGFR, two targets that may be associated with toxicities such as increased myelosuppression and pleural effusion, respectively. Through its dual inhibition, bosutinib is a more potent inhibitor of the Bcr-Abl tyrosine kinase than imatinib in untreated CML patients (Remsing Rix et al., 2009).

## Bosutinib Trial Results

Data from a phase I/II study demonstrated efficacy and acceptable tolerability in 118 patients with CP-CML who had resistance or intolerance to prior TKI therapy (imatinib, dasatinib, and/or nilotinib). After a 28.5-month follow-up, major cytogenetic response (MCyR; see Table 1) and CCyR were attained in 32% and 24% of patients, respectively. The estimated 2-year PFS was 73%, and the estimated OS was 83%. Hematologic and cytogenetic responses to bosutinib were observed in patients with mutations within the Bcr-Abl kinase domain except for the T315I mutation. During follow-up, five (4%) patients experienced a confirmed transformation to AP, and no transformations to BP occurred (Khoury et al., 2012).

**Table 1:**
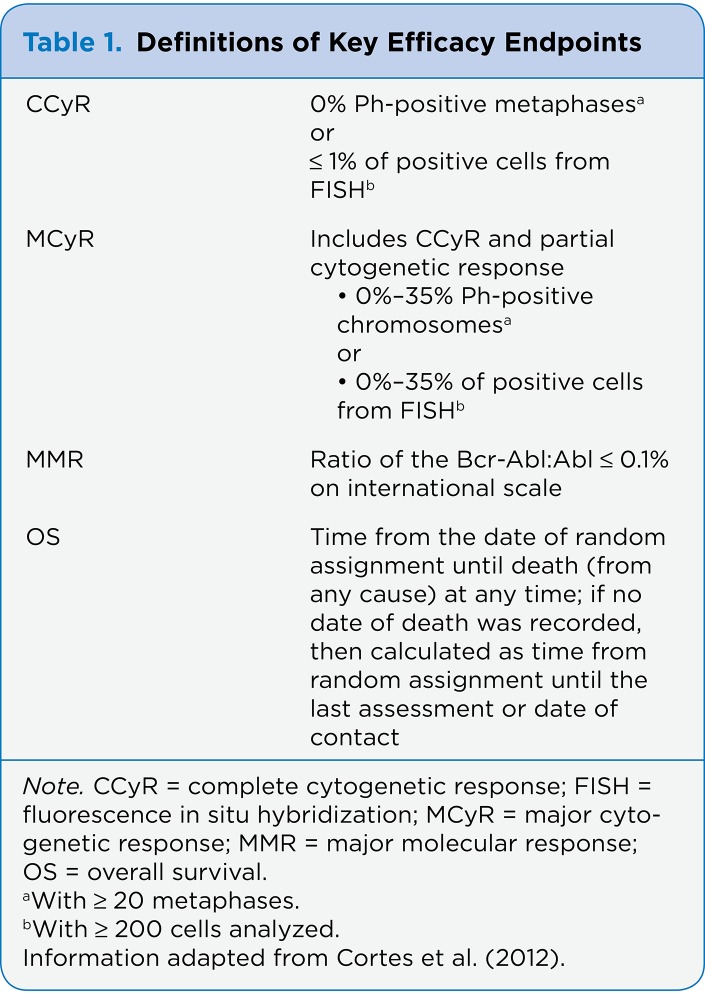
Table 1. Definitions of Key Efficacy Endpoints

In the phase III BELA (Bosutinib Efficacy and Safety in Newly Diagnosed Chronic Myeloid Leukemia) trial comparing bosutinib with imatinib in the first-line setting, bosutinib did not achieve the primary efficacy endpoint of improved CCyR at 12 months. The CCyR rate at 12 months was not different for bosutinib (70%; 95% confidence interval [CI] = 64%–76%) vs. imatinib (68%; 95% CI = 62%–74%; two-sided* p* = .601; Cortes et al., 2012). In addition, bosutinib was associated with a higher rate of discontinuation due to adverse effects compared with imatinib (19% vs. 6%;* p* = .601). However, bosutinib demonstrated superiority in several secondary endpoints when compared with imatinib.

The major molecular response (MMR) rate at 12 months was higher for bosutinib (41%; 95% CI = 35%–47%) vs. imatinib (27%; 95% CI = 22%–33%; * p* < .001). The times to CCyR and MMR were faster with bosutinib (* p* < .001), which may provide a more rapid debulking of tumor burden compared with imatinib (Cortes et al., 2012). A previous study with imatinib in newly diagnosed CML showed a correlation with MMR at 12 months, including better PFS (99% vs. 94% with no MMR;* p* = .0023) and improved OS (99% vs. 93% with no MMR;* p* = .0011) at 3 years (Hehlmann et al., 2011). Further follow-up is currently ongoing to determine whether the faster times to CCyR and MMR with bosutinib will correlate with long-term duration of response and improvement in outcomes (PFS or OS).

Bosutinib offers an additional treatment option for patients with Bcr-Abl mutations. In murine myeloid cell lines, bosutinib demonstrated inhibition against 16 of 18 imatinib-resistant forms of Bcr-Abl. Specifically, bosutinib did not inhibit T315I and V299L mutations (Pfizer, 2012). Further, bosutinib has demonstrated clinical response in patients resistant or intolerant to dasatinib or nilotinib therapy. Patients with mutations linked to resistance with dasatinib (F317L) and nilotinib (Y253H and F359C/I/V) were among those who responded to bosutinib (Khoury et al., 2012).

## Dosing and Administration

The recommended dose of bosutinib is 500 mg given orally once daily with food. Advanced practitioners should advise patients to take bosutinib with a high-fat meal, as it will improve absorption and tolerability (Abbas et al., 2011). Treatment is continued until the occurrence of intolerance or progression of disease. For patients without grade 3 or greater adverse reactions who do not have a complete hematologic response by week 8 or CCyR by week 12, advanced practitioners should consider a dose increase to 600 mg daily. An initial dose reduction to 200 mg given orally once daily is recommended for patients with baseline mild, moderate, or severe hepatic impairment (see Table 2). No renal dose adjustment is necessary based on pharmacokinetic studies in subjects with creatinine clearance ranging from 25 to 120 mL/min (Pfizer, 2012).

**Table 2:**
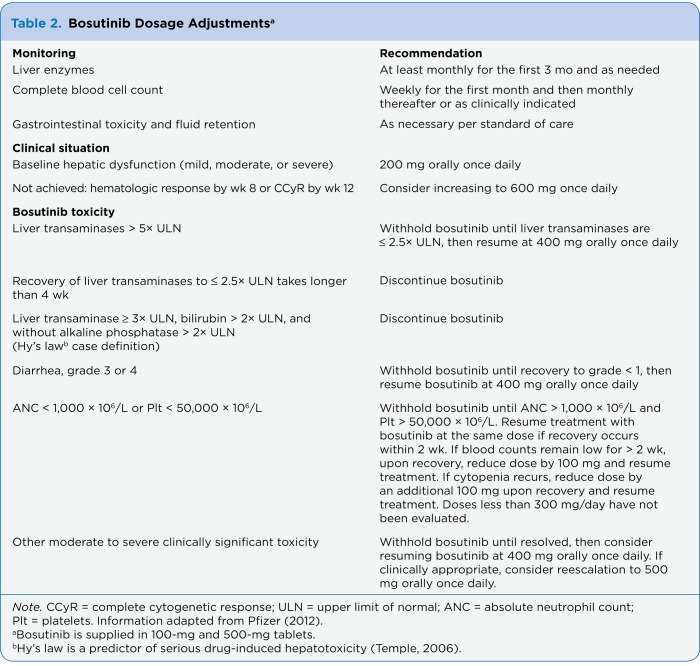
Table 2. Bosutinib Dosage Adjustments

Specific dose adjustments for bosutinib toxicities such as elevated liver transaminase levels, diarrhea, neutropenia, and thrombocytopenia are included in Table 2 (Pfizer, 2012). For other clinically significant toxicities, bosutinib should be held until the toxicity resolves and subsequently restarted at 400 mg orally daily. Reescalation to 500 mg orally daily may be considered if clinically appropriate (Pfizer, 2012). In the BELA trial, a patient who required a dose reduction due to nonhematologic toxicity could be considered for reescalation if he or she tolerated the reduced dose for at least 1 month with no toxicity (Cortes et al., 2012).

Bosutinib is primarily metabolized by CYP3A4 to inactive metabolites. Renal clearance is negligible (only 3% of a radiolabeled dose was recovered in the urine of healthy volunteers). Strong/moderate CYP3A4 inhibitors, strong/moderate CYP3A4 inducers, and P-glycoprotein (P-gp) inhibitors should be avoided (see Table 3). In vitro and in vivo studies suggest bosutinib absorption is pH-dependent. In one study, use of a proton pump inhibitor (PPI) decreased maximum concentration (Cmax) by 46% and the area under the curve (AUC) by 26%. If acid reduction is necessary, the advanced practitioner should consider replacing the PPI with a short-acting antacid or H2 receptor antagonist and separating administration of any antacid products from bosutinib administration by at least 2 hours (Pfizer, 2012).

**Table 3:**
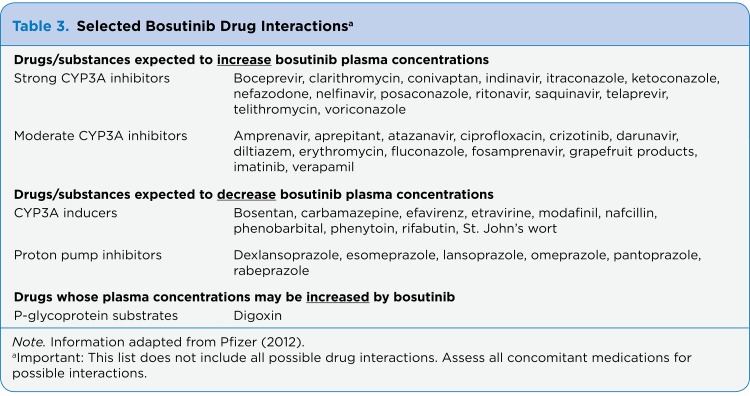
Table 3. Selected Bosutinib Drug Interactions

## Adverse Events

Frequent adverse effects of any grade reported in the phase I/II study (safety population, n = 546) included diarrhea (82%), nausea (46%), thrombocytopenia (41%), vomiting (39%), abdominal pain (37%), rash (35%), anemia (27%), pyrexia (26%), and fatigue (24%). Median time to onset of diarrhea was 2 days, median duration per event was 1 day, and median number of episodes among those who experienced diarrhea was 3. Grade 3/4 myelosuppression occurred more frequently in patients in AP or BP compared with those with CP-CML (thrombocytopenia 57% vs. 25%, neutropenia 37% vs. 18%, and anemia 35% vs. 13%, for AP/BP vs. CP-CML, respectively). Based on reports of hepatic toxicity in clinical trials, advanced practitioners should conduct hepatic enzyme tests monthly for the first 3 months of bosutinib therapy (Pfizer, 2012).

The toxicity profile of bosutinib differs from that of other TKIs. Specifically, in the BELA study, bosutinib was associated with a higher incidence of diarrhea, vomiting, and abdominal pain than imatinib but a lower incidence of edema, bone pain, and muscle spasms (Cortes et al., 2012).

## Conclusion

Although the past 2 decades have seen significant improvement in the outcomes for patients with CML due to the advent of targeted therapy against Bcr-Abl, there is still a need for further treatment options for those patients who have progressed to more advanced phases of disease as a result of drug resistance. To date, a recommendation does not exist for a treatment algorithm when choosing a second- or third-line TKI. Advanced practitioners must consider both mutation profile and tolerability when choosing an agent.

Bosutinib is a recently approved TKI indicated for the treatment of adult patients with Ph+ CML who are resistant or intolerant to prior therapy. This agent has activity against many mutations resistant to other TKIs but has poor activity against the T315I and V299L mutations (Redaelli et al., 2009; Pfizer, 2012). Bosutinib has activity against Src and Abl kinases and is more potent than imatinib. It was associated with a toxicity profile distinct from imatinib with greater rates of diarrhea, vomiting, and aminotransferase elevations. Long-term follow-up data are needed to determine duration of response, transformation rate to AP/BP-CML, and OS.
